# Retracted and Republished from: “Gut Microbiota Mediates the Therapeutic Effect of Monoclonal Anti-TLR4 Antibody on Acetaminophen-Induced Acute Liver Injury in Mice”

**DOI:** 10.1128/spectrum.04715-22

**Published:** 2023-03-21

**Authors:** Xuewei Sun, Qian Cui, Juan Ni, Xiaoguang Liu, Jin Zhu, Tingting Zhou, HuaYing Huang, Ke OuYang, Yulong Wu, Zhan Yang

**Affiliations:** a Centre for Diseases Prevention and Control of Eastern Theater, Nanjing, China; b Binzhou Medical University, Yantai, China; c Air Force Hospital of Eastern Theater, Nanjing, China; d The First Affiliated Hospital of Nanjing Medical University (Jiangsu Province Hospital), Nanjing, China; e Department of Respiratory and Critical Care Medicine, Jinling Hospital, The First School of Clinical Medicine, Southern Medical University, Nanjing, China; University of Nebraska—Lincoln

**Keywords:** gut microbiota, acetaminophen, acute liver injury, TLR4

## Abstract

Acetaminophen (APAP) overdose is one of the most common causes of acute liver injury (ALI) in Western countries. Many studies have shown that the gut microbiota plays an important role in liver injury. Currently, the only approved treatment for APAP-induced ALI is *N*-acetylcysteine; therefore, it is essential to develop new therapeutic agents and explore the underlying mechanisms. We developed a novel monoclonal anti-Toll-like receptor 4 (TLR4) antibody (ATAB) and hypothesized that it has therapeutic effects on APAP-induced ALI and that the gut microbiota may be involved in the underlying mechanism of ATAB treatment. Male C57BL/6 mice were treated with APAP and ATAB, which produced a therapeutic effect on ALI and altered the members of the gut microbiota and their metabolic pathways, such as *Roseburia*, *Lactobacillus*, *Akkermansia*, and the fatty acid pathway, etc. Furthermore, we verified that purified short-chain fatty acids (SCFAs) could alleviate ALI. Moreover, a separate group of mice that received feces from the ATAB group showed less severe liver injury than mice that received feces from the APAP group. ATAB therapy also improved gut barrier functions in mice and reduced the expression of the protein zonulin. Our results revealed that the gut microbiota plays an important role in the therapeutic effect of ATAB on APAP-induced ALI.

**IMPORTANCE** In this study, we found that a monoclonal anti-Toll-like receptor 4 antibody can alleviate APAP-induced acute liver injury through changes in the gut microbiota, metabolic pathways, and gut barrier function. This work suggested that the gut microbiota can be a therapeutic target of APAP-induced acute liver injury, and we performed foundation for further research.

## INTRODUCTION

Acetaminophen (APAP) is one of the most widely used analgesic antipyretics (1), and its overdose is the main cause of acute liver injury (ALI) in Western countries (2, 3). However, effective drugs to treat ALI are lacking, and *N*-acetylcysteine (NAC) is the only approved drug for patients who overuse APAP (4, 5), but the application timing is restricted. Therefore, there is an urgent need to develop new drugs and explore their molecular mechanisms.

Pattern recognition receptors are germ line-encoded, evolutionarily conserved receptors that recognize microbe-associated molecular patterns and damage-associated molecular patterns (6). Toll-like receptor 4 (TLR4) is one of the first identified pattern recognition receptors and plays an important role in the innate immune response (7). TLR4 also plays an important role in ALI. For example, fetuin-A, a hepatokine, is involved in APAP-induced ALI through the activation of TLR4 (8), and TLR4 antagonism can reduce proinflammatory gene expression (9). Kupffer cells can be activated by TLR4/MyD88 signaling in ALI; TLR4 knockout (TLR4-KO) mice were protected from liver necrosis; and similarly, STM28, a TLR4 antagonist, attenuated liver injury and necrosis and reduced creatinine levels (10–12). Evidence shows that agents that can alter the immune response can be developed into therapeutic drugs (13, 14).

TLR4 also plays an important role in gut homeostasis and disease (15); for example, the disruption of TLR signaling increases epithelial cell proliferation in the transit-amplifying zone (16), and the activation of TLR4 in intestinal stem cells reduces proliferation and enhances apoptosis in the small intestine and colon organoids (17, 18). Enhanced epithelial TLR4 signaling is associated with impaired epithelial barrier function and microbiota changes (19) and can increase intestinal permeability via an intracellular mechanism involving the TLR4-dependent upregulation of CD14 membrane expression and myosin light chain kinase expression (20, 21). The gut microbiota and gut barrier are also associated with liver disease. In recent years, much attention has been focused on the reciprocal interaction between the gut and the liver. This interaction is termed the gut-liver axis, which refers to the bidirectional relationship between the gut, along with its microbiota, and the liver (22, 23). Several liver diseases are closely related to the gut-liver axis (24–27). For example, Nlrp6^−/−^ mice, a model for gut dysbiosis, showed severe liver injury induced by APAP or lipopolysaccharides (LPSs) (22), which is associated with the early onset of increased intestinal permeability and bacterial translocation (28).

Therefore, we developed a monoclonal anti-TLR4 antibody (ATAB) in our lab that can bind to TLR4 with a high affinity and inhibit LPS-induced TLR4 signaling (29) and verified that the ATAB had a therapeutic effect on APAP-induced ALI (30), and now, we hypothesize that the gut microbiota may also mediate this effect. Therefore, the present study aimed to determine the therapeutic effects of ATAB on APAP-induced ALI and examine the role of the gut microbiota involved.

## RESULTS

### APAP-induced ALI was alleviated by ATAB.

**FIG 1:**
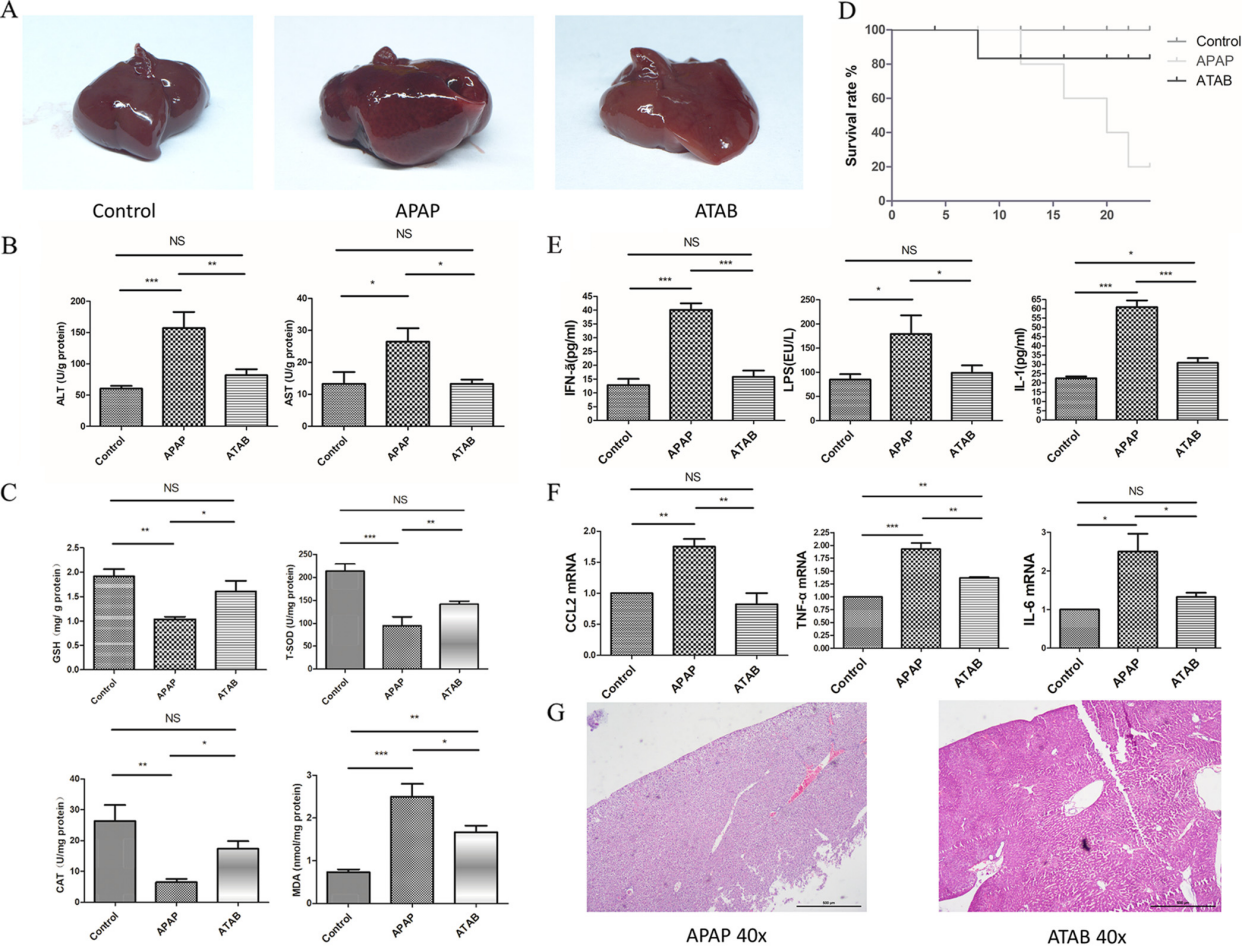
ATAB can alleviate APAP-induced acute liver injury (ALI). (A) Liver congestion is more serious in the APAP group than in the ATAB group. (B) The plasma concentrations of ALT and AST are much lower in the ATAB group (*n* = 7 for each group). (C) The ATAB group displayed liver oxidative stress indices (MDA, SOD, GSH, and CAT) different from those of the APAP group (*n* = 8 for each group). (D) ATAB improved survival from ALI (*n* = 8 for each group). (E and F) The ATAB group exhibited low levels of inflammatory cytokines, LPS, and chemokines in plasma and liver tissues (*n* = 7 for each group). IFN-ã, interferon alpha. (G) HE staining revealed less necrosis of liver cells in the ATAB group than in the APAP group. The data are presented as the means ± SEM. *, *P* < 0.05; **, *P* < 0.01; ***, *P* < 0.001; NS, not significant (for the comparisons).

Mice were treated with APAP (600 mg/kg of body weight) for ALI and then treated with ATAB or phosphate-buffered saline (PBS) as a control. We found that the ATAB group showed mild liver damage compared with the APAP group. Liver congestion was more serious in the APAP group than in the ATAB group (Fig. 1A). Plasma alanine aminotransferase (ALT) and aspartate aminotransferase (AST) levels were much lower in the ATAB group than in the APAP group (Fig. 1B). Besides, the malondialdehyde (MDA) level was lower in the ATAB group than in the APAP group, but superoxide dismutase (SOD), glutathione (GSH), and catalase (CAT) levels were higher in the ATAB group than in the APAP group (Fig. 1C). ALI may induce death in a mouse model, and we found that ATAB improved the survival rate (Fig. 1D). After the mice were sacrificed, inflammatory cytokines, lipopolysaccharide (LPS), and chemokines were detected in the plasma and liver tissues, and low expression levels were observed in both sample types (Fig. 1E and F). To observe liver injury, hematoxylin and eosin (HE) staining of the liver tissue was performed, which revealed reduced liver cell necrosis in the ATAB group (Fig. 1G).

### ATAB altered the gut microbiota composition during ALI treatment.

First, we used a 16S rRNA sequencing method to identify the changes in the gut microbiota after APAP overuse. Besides the control group, we chose two time points to detect the gut microbiota, 6 h and 24 h after mice were treated with APAP (APAP 6-h group and APAP 24-h group). Principal-coordinate analysis (PCoA) and nonmetric multidimensional scaling (NMDS) methods showed that the gut microbiota structures were different among the three groups (see Fig. S1 in the supplemental material), and the heat map at the genus and phylum levels also showed changes (Fig. S2).

**FIG 2:**
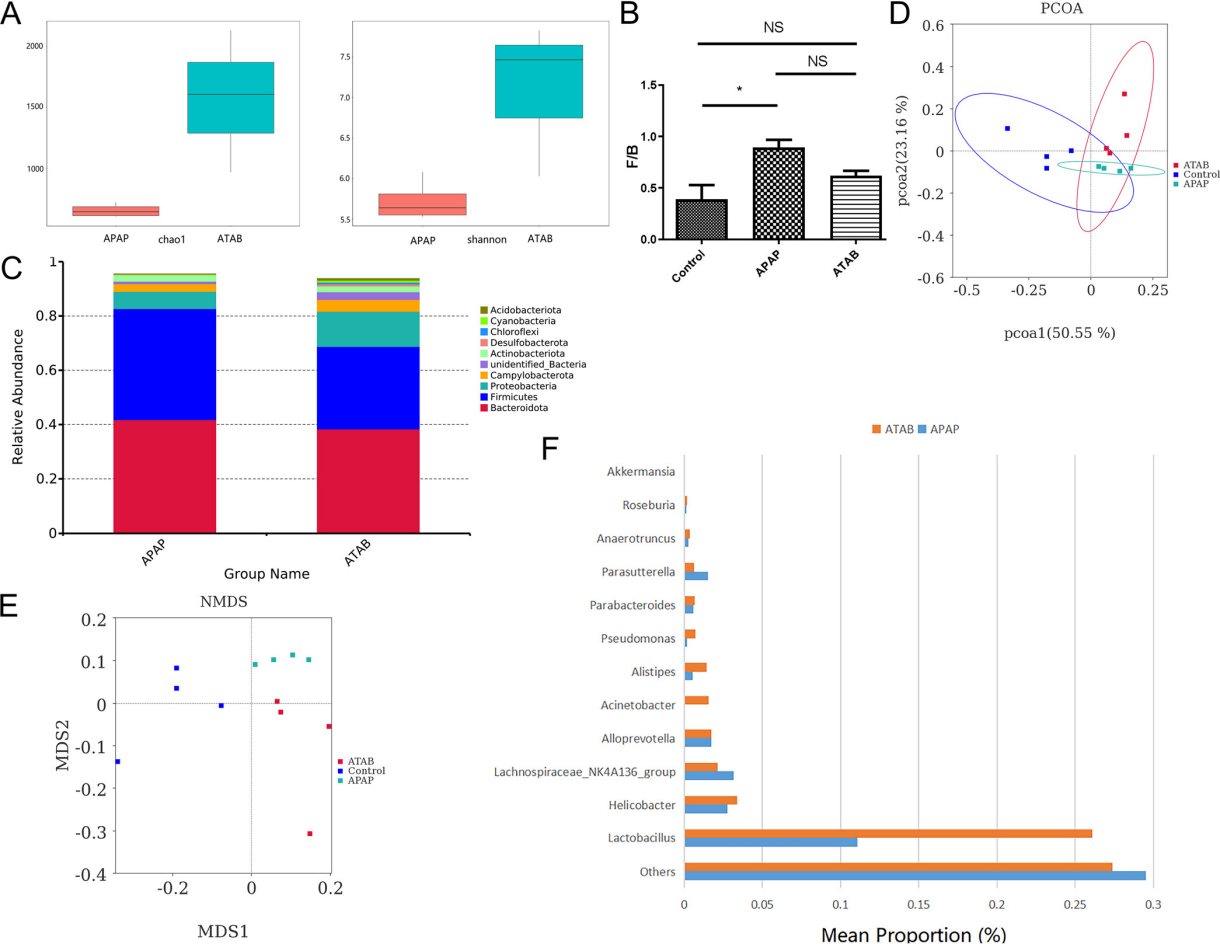
ATAB altered the gut microbiota composition during ALI treatment. (A) The ATAB group had higher Shannon diversity indices and Chao1 indices. (B and C) The APAP group showed a higher F/B ratio of the gut microbiota, showing that the composition at the phylum level differs between the two groups. The ATAB group has lower *Firmicutes* and *Bacteroidetes* levels, but the level of *Proteobacteria* appears to be higher than that in the APAP group. (D and E) PCoA and NMDS analysis of the gut microbiota. These results show significant differences among the APAP, ATAB, and control groups. (F) The ATAB group had higher abundances of some bacterial genera such as *Roseburia*, *Lactobacillus*, and *Akkermansia* (*n* = 3 to 4 for each group). The data are presented as the means ± SEM. *, *P* < 0.05; **, *P* < 0.01; ***, *P* < 0.001 (for the comparisons). MDS, metric multi-dimensional.

To explore the influence of ATAB, the composition of the gut microbiota was evaluated. We analyzed the Shannon diversity index and the Chao1 index. The ATAB group had higher values than the APAP group (Fig. 2A). These results indicated that the gut microbiota diversity in the APAP-induced ALI mice had changed and that ATAB could enhance the biodiversity of the gut microbiota. We also examined the *Firmicutes*/*Bacteroidetes* (F/B) ratio and the composition of the gut microbiota at the phylum level. These results showed that the APAP group had a higher F/B ratio than the ATAB group. Besides, the compositions at the phylum level differed between the two groups. The ATAB group had lower *Firmicutes* and *Bacteroidetes* proportions, but *Proteobacteria* proportions were higher than those in the APAP group (Fig. 2B and C). Next, we analyzed the gut microbiota composition using PCoA and NMDS methods (Fig. 2D and E). These results revealed significant differences among the APAP, ATAB, and control groups, indicating that the use of ATAB can alter the gut microbiota composition. The ATAB group had higher abundances of some bacterial genera such as *Roseburia*, *Lactobacillus*, and *Akkermansia* (Fig. 2F).

### ATAB altered the gut microbiota metabolome during ALI treatment.

**FIG 3:**
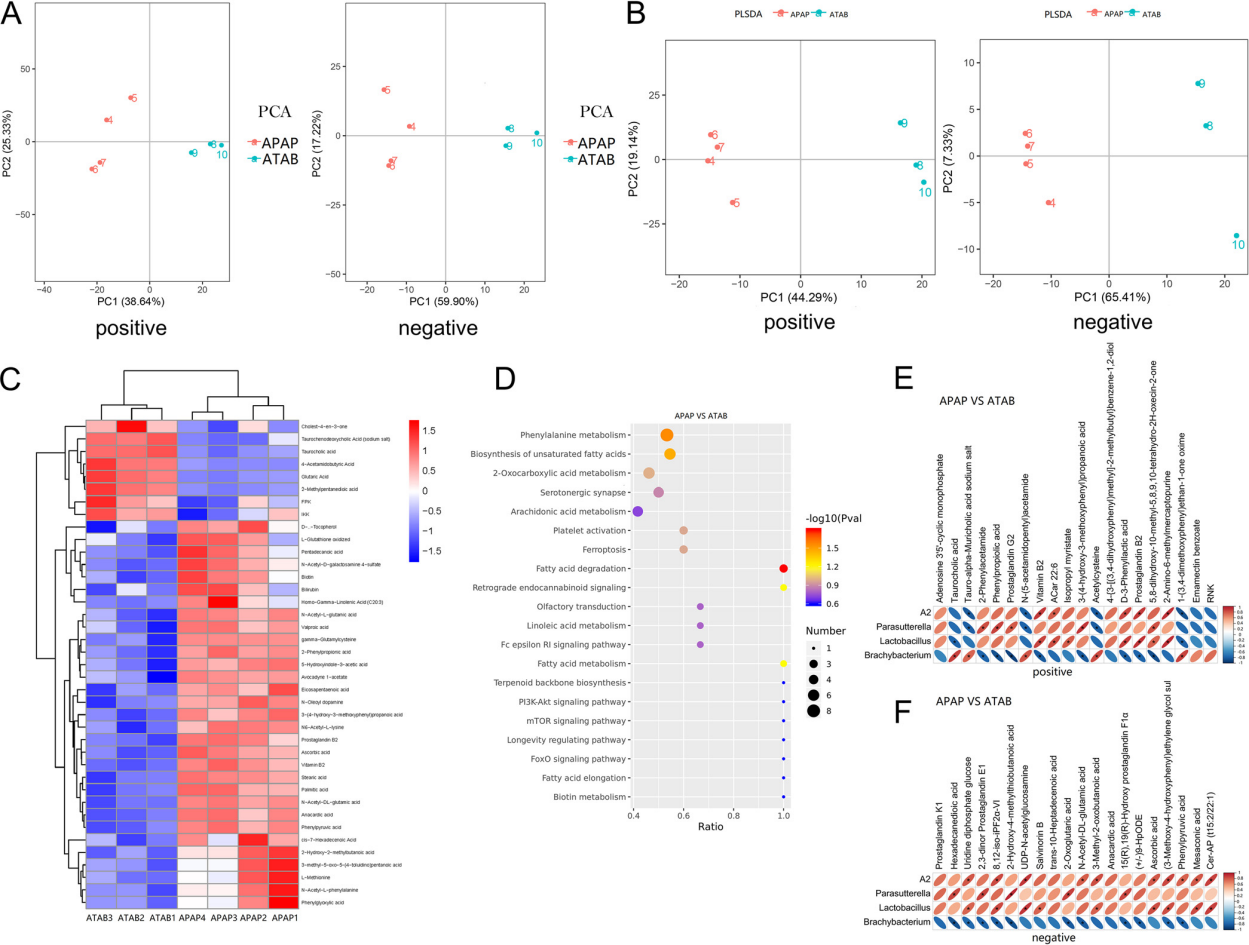
ATAB alters the metabolome of the gut microbiota. (A and B) PCA and PLS-DA show a significant difference in the metabolic profiles of the ATAB and APAP groups (the metabolites are separated by positive and negative charges). (C) Heatmap showing metabolite differences between the two groups. (D) Various metabolic pathways, including fatty acid degradation, linoleic acid metabolism, fatty acid metabolism, fatty acid elongation, the biosynthesis of unsaturated fatty acids, retrograde endocannabinoid signaling, biotin metabolism, the PI3K-Akt signaling pathway, and the mTOR signaling pathway. (E and F) Conjoint analyses of 16S rRNA sequences and the metabolome show key bacteria and metabolites as well as potential correlations (the metabolites are separated by positive and negative charges) (*n* = 3 to 4 for each group). IKK, inhibitor of kappa B kinase; FPK, fructose-phosphokinase; iPF2α, isoprostaglandin F2α; HpODE, hydroperoxyoctadeca-10,12-dienoic acid; Cer-AP, caerulein-AP; RNK, the oligopeptide of arginine, asparagine, and lysine.

A metabolomic analysis was performed using liquid chromatography-mass spectrometry (LC-MS) to determine whether ATAB can change the gut microbiota metabolome. More than a thousand metabolites were identified, including sugars, amino acids, fatty acids, and organic acids. These compounds are involved in metabolism, genetic information processing, environmental information processing, cellular processes, and organismal systems. Principal-component analysis (PCA) and partial least-squares discriminant analysis (PLS-DA) were used to identify metabolites (we separated the positively and negatively charged metabolites) and revealed that the APAP group displayed metabolic profiles that were significantly different from those of the ATAB group (Fig. 3A and B). A heatmap (Fig. 3C) revealed the differences in the metabolites between the two groups. The different metabolites included taurochenodeoxycholic acid, glutaric acid, 2-methylpentanedioic acid, pentadecanoic acid, *N*-acetyl-l-glutamic acid, 3-(4-hydroxy-3-methoxyphenyl)propanoic acid, biotin, and vitamin B_2_. We then analyzed the metabolic pathways (Fig. 3D), which showed fatty acid-relevant metabolic pathways such as fatty acid degradation, linoleic acid metabolism, fatty acid metabolism, fatty acid elongation, and the biosynthesis of unsaturated fatty acids. Other noteworthy pathways included retrograde endocannabinoid signaling, biotin metabolism, the phosphatidylinositol 3-kinase (PI3K)-Akt signaling pathway, and the mTOR signaling pathway.

Based on 16S rRNA sequencing and metabolome analysis, we performed a conjoint analysis, which revealed key bacteria, key metabolites, and their potential correlations; for example, *Lactobacillus*, a key bacterial genus, was correlated with isopropyl myristate, vitamin B_2_, and taurocholic acid, etc. (we separated the positively and negatively charged metabolites). (Fig. 3E and F). These results provided us with many target bacteria and their metabolites for conducting further research.

### The gut microbiota of the ATAB group alleviated ALI via fecal microbiota transplantation.

To further analyze the role of the gut microbiota in the progression of ATAB treatment, fecal microbiota transplantation (FMT) was performed. The feces of the ATAB and APAP groups were orally administered to recipients whose gut microbiota had been depleted with broad-spectrum antibiotics (ABX) for 5 days. The gut microbiota of the recipients was similar to that of the donors, indicating that the FMT was successful. After 3 days, the mice were treated with 600 mg/kg APAP and sacrificed 24 h after APAP treatment.

**FIG 4:**
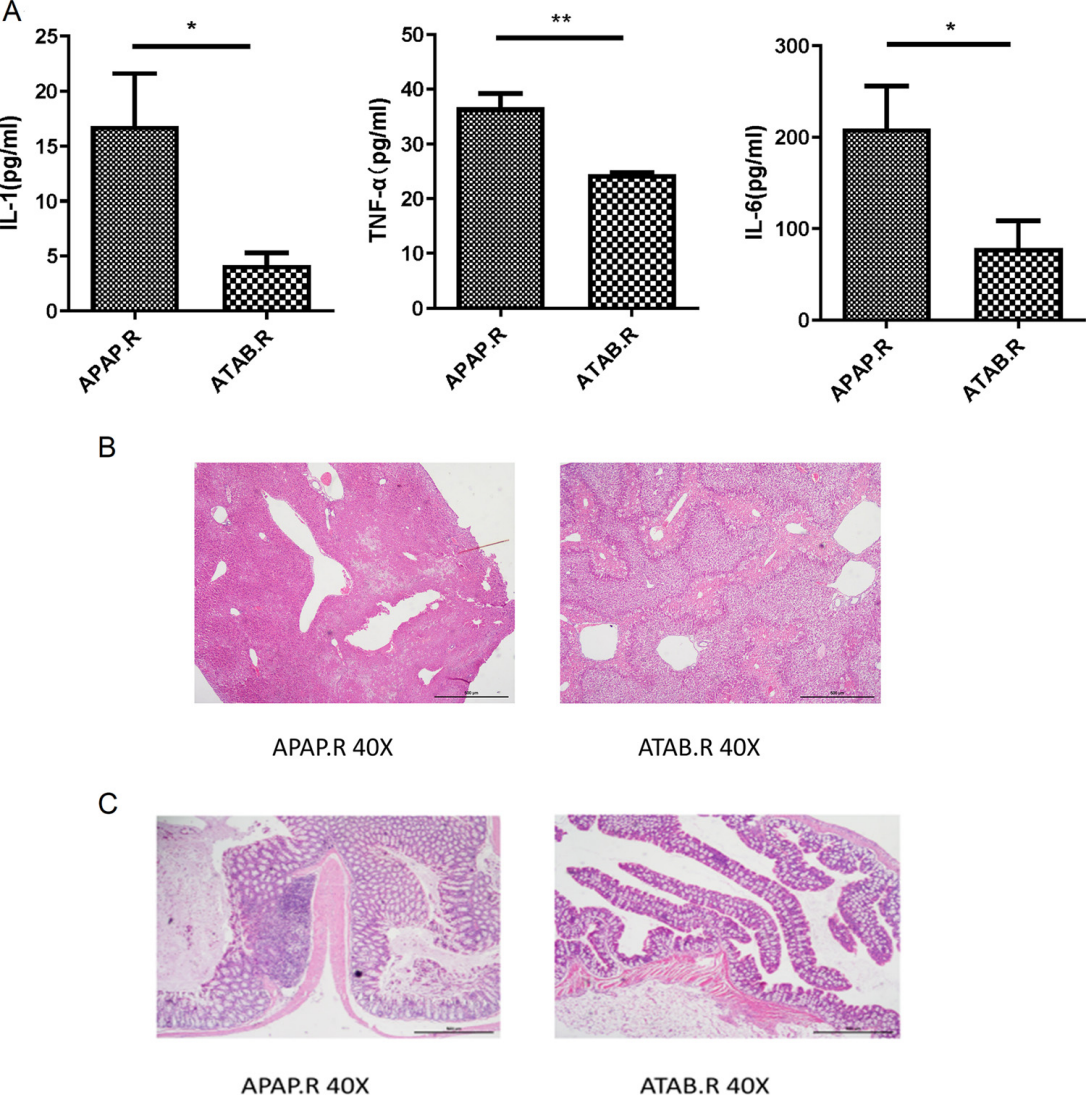
The gut microbiota from the ATAB group can alleviate ALI through FMT. (A) Mice that received ATAB feces had lower IL-1, TNF-α, and IL-6 levels than the APAP group. (B) HE staining shows that ALI in mice receiving feces from the ATAB group was less severe than that observed in mice receiving feces from the APAP group. (C) The colon tissues of mice receiving feces from the APAP group display inflammatory cell infiltration, but those of the mice receiving feces from the ATAB group show no obvious changes (*n* = 4 to 5 for each group). The data are presented as the means ± SEM. *, *P* < 0.05; **, *P* < 0.01; ***, *P* < 0.001 (for the comparisons).

The mice that received feces from the ATAB group had lower interleukin-1 (IL-1), tumor necrosis factor alpha (TNF-α), and IL-6 levels than the APAP group (Fig. 4A). HE staining showed that ALI in the recipients of the feces from the ATAB group was less severe than that in the recipients of feces from the APAP group (Fig. 4B). However, the amelioration of ALI was not as evident as that observed with the direct administration of ATAB. Moreover, the colon tissues of recipients of feces from the APAP group showed inflammatory cell infiltration (Fig. 4C), indicating that these feces may contribute to inflammation.

### ATAB promoted gut barrier function compared with APAP administration.

**FIG 5:**
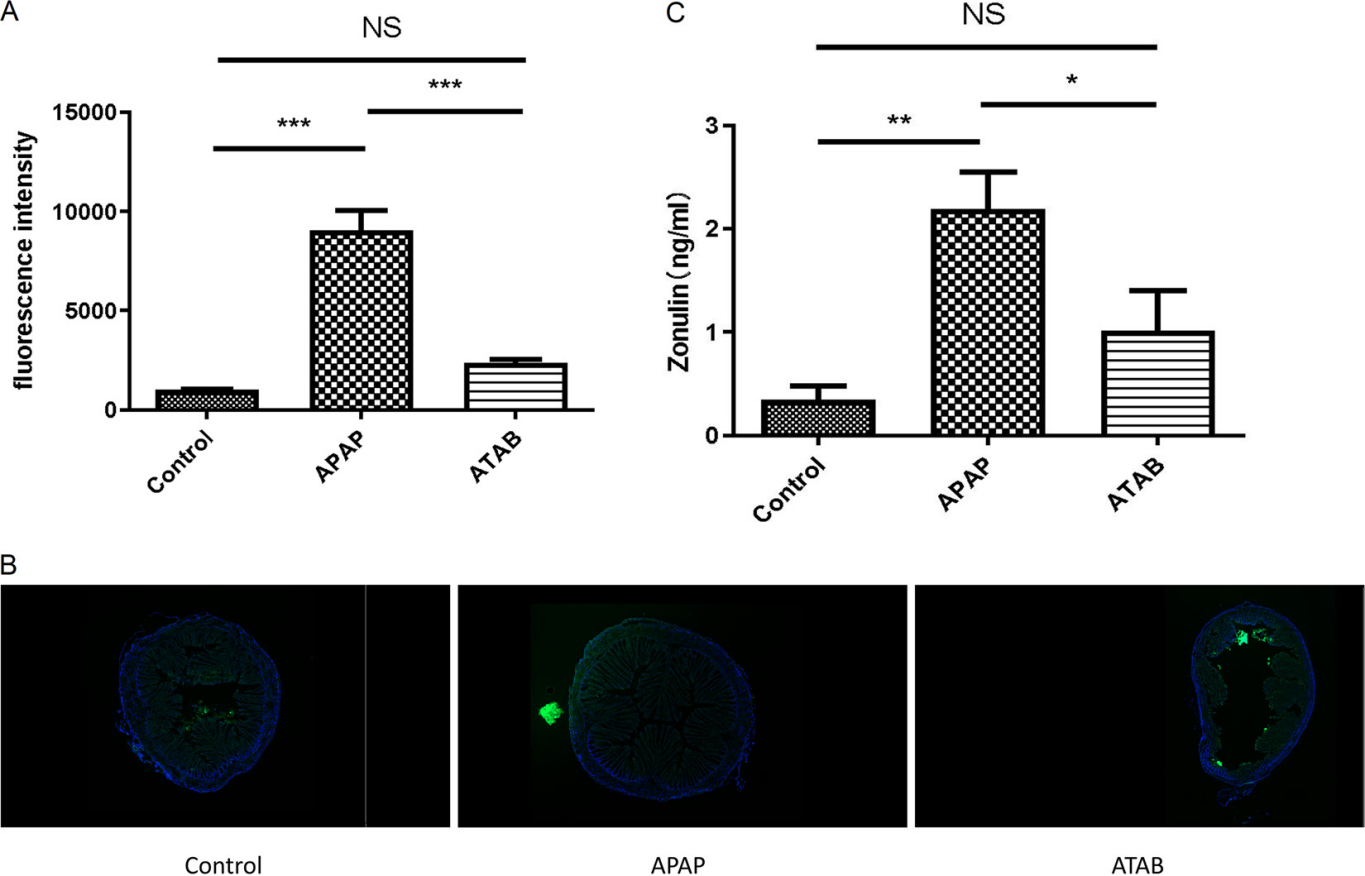
ATAB can promote gut barrier functions. (A) The APAP group shows higher fluorescence intensity in the serum than the ATAB group. (B) In the colon tissues, the APAP group displays FITC-dextran extravasation, while FITC-dextran in the ATAB group was confined mainly to the colon lumen. (C) The ATAP group displayed higher levels of the protein zonulin than the ATAB group (*n* = 3 for each group). The data are presented as the means ± SEM. *, *P* < 0.05; **, *P* < 0.01; ***, *P* < 0.001 (for the comparisons).

We used fluorescein isothiocyanate (FITC)-dextran oral administration to detect gut barrier function. Approximately 20 h after APAP injection, mice were administered 4-kDa FITC-dextran (FD4) orally, blood samples were collected after 4 h, and the fluorescence intensity of FITC-dextran in the peripheral blood was measured. The APAP group showed a high fluorescence intensity, whereas the ATAB group showed a lower fluorescence intensity in the serum (Fig. 5A). We also observed colon tissues, and the APAP group showed extravasation of FITC-dextran, whereas the ATAB group showed confinement of FITC-dextran to the colon lumen (Fig. 5B). As zonulin is a modulator of intestinal epithelial cell tight junctions, we quantified the protein content in the colon tissues of mice from the APAP and ATAB groups using an enzyme-linked immunosorbent assay (ELISA). The results revealed that the APAP group had higher zonulin levels than the ATAB group (Fig. 5C). Therefore, our results demonstrated that ATAB can promote gut barrier function.

### Short-chain fatty acids can alleviate APAP-induced ALI without ATAB.

**FIG 6:**
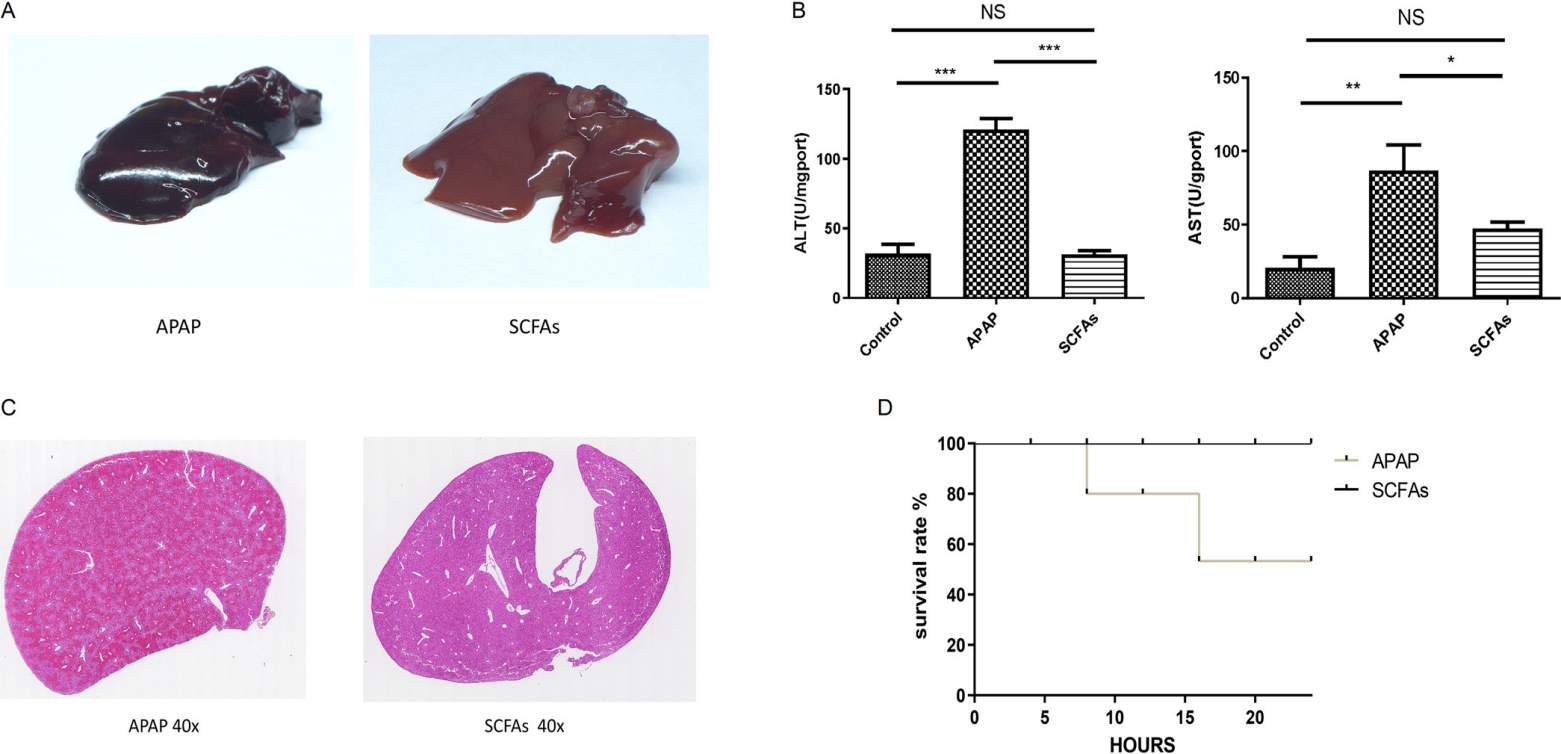
Short-chain fatty acids (SCFAs) can alleviate APAP-induced ALI without ATAB. (A) Liver congestion was milder in the SCFA group. (B) Plasma ALT and AST levels were lower in the SCFA group. (C) HE staining shows decreased liver cell necrosis in the SCFA group. (D) The survival rate was 100% in the SCFA group, compared with 40% in the APAP group (*n* = 5 to 7 for each group). The data are presented as the means ± SEM. *, *P* < 0.05; **, *P* < 0.01; ***, *P* < 0.001 (for the comparisons).

Metabolomic analysis revealed that the fatty acid metabolic pathways differed between the two groups, so well-established metabolites, short-chain fatty acids (SCFAs), were chosen to determine whether they might influence APAP-induced ALI. The SCFAs were dissolved in water and given to mice for free drinking for 7 days. Thereafter, APAP was injected, and the blood and liver tissues of mice were collected after 24 h. The results showed that liver congestion was milder (Fig. 6A) and plasma ALT and AST levels were much lower in the SCFA group (Fig. 6B) than in the control group. HE staining showed reduced liver cell necrosis in the SCFA group (Fig. 6C). Besides, the survival rate was 100% in the SCFA group but only 40% in the APAP group (Fig. 6D). The treatment effect of SCFAs was evident as they alleviated APAP-induced ALI without ATAB administration.

## DISCUSSION

In this study, we investigated the therapeutic effect of ATAB on APAP-induced ALI and analyzed the role of the gut microbiota, gut metabolism, and the gut barrier in this process.

The ATAB in our experiment is TLR4 IgG2 antibody (30). There are four subclasses of IgG (IgG1, IgG2, IgG3, and IgG4), which are defined by their unique structural and functional characteristics (31). IgG1 has functions in antibody-dependent cellular cytotoxicity (ADCC) and apoptosis induction (32–34). IgG4 is a neutralizing inhibitory signal in T cells (35–37). IgG3 has a long hinge region and a polymorphic nature; it increases the risk of stability and immunogenicity (38, 39). Thus, they were inappropriate for the development of a TLR4 antibody, and we chose IgG2 to develop the ATAB.

We found that ATAB treatment altered the composition of the gut microbiota, including the F/B ratio, the composition at the phylum level. We observed that specific bacteria differed between the ATAB and APAP groups, such as *Lactobacillus*, *Akkermansia*, and *Roseburia*. *Lactobacillus* is a well-studied probiotic, and some specific strains are useful for treating liver disease; for example, Lactobacillus rhamnosus LGG can ameliorate liver injury and hypoxic hepatitis in a rat model of CLP (cecal ligation and puncture)-induced sepsis (40), Lactobacillus plantarum CMU995 can ameliorate alcohol-induced liver injury by improving both the intestinal barrier and antioxidant activity (41), and Lactobacillus acidophilus LA14 alleviates liver injury induced by d-galactosamine (d-GalN) in rats (42). ATAB may increase the abundance of *Lactobacillus* in the gut microbiota and play a role in the alleviation of ALI. Akkermansia muciniphila, a novel probiotic, has recently attracted increasing attention. A recent study showed that Akkermansia muciniphila can protect mice from high-fat diet (HFD)/CCl_4_-induced liver injury (43). In our study, ATAB enhanced *Akkermansia* levels, which may contribute to the therapeutic effect. *Roseburia* is another bacterial genus that is more abundant in the ATAB group. *Roseburia* can ameliorate alcoholic fatty liver in mice (44) and is the source of propionate and butyrate in the gut (45–47). Therefore, these bacteria may have different probiotic functions.

Interestingly, the change in the gut microbiota lasted for a longer time than expected, as some studies had confirmed that centrilobular necrosis occurred within 6 to 12 h after APAP overuse (48), but our results showed that 6 h after APAP overuse, the gut microbiota had changed, and after 24 h of APAP overuse, the gut microbiota was continually changing. This showed that the change in the gut microbiota is different from the necrosis and apoptosis of hepatocytes. So the therapeutic modality targeting the gut microbiota may have a longer therapeutic window.

FMT research is necessary for microbiome studies to transform correlation to causation (49); therefore, we performed FMT to evaluate the exact function of the gut microbiota. Feces from the ATAB group can alleviate ALI, although the therapeutic effect is not as obvious as that of ATAB, suggesting that the gut microbiota and metabolites mediate the therapeutic effect of ATAB, at least in part. Thus, ATAB may also function via other underlying mechanisms that treat APAP-induced ALI.

The importance of metabolites in gut microbiota studies is acknowledged; they exert their effects as signaling molecules and substrates for metabolic reactions (50). In this study, we found that several metabolic pathways differ between the ATAB and APAP groups, such as fatty acid- and bile acid-relevant metabolic pathways. The synthesis and secretion of bile acids are two of the most important functions of the liver, and they influence the gut-liver axis. Lipopolysaccharides from gut bacteria can enter the liver through the portal circulation, which can combine with TLR4 and aggravate the inflammatory response, causing liver cell death (51). Therefore, this may also represent the mechanism by which ATAB alleviates liver injury.

SCFAs are the products of bacteria in the cecum and colon (52), have many beneficial effects (47), and are also involved in fatty acid metabolism. However, there have been few studies on SCFAs and liver injury. We provided SCFAs directly to APAP-treated mice and found that SCFAs alleviated ALI unexpectedly. Metabolites of the gut microbiota influence physical health; therefore, we performed metabolomic analysis to reveal multiple metabolites to be further explored as research targets.

Intestinal barrier function is associated with intestinal and extraintestinal diseases, including liver disease (27, 53). APAP increases intestinal permeability, which may explain the induction of ALI but not the direct cytotoxicity to liver cells (54, 55). We found that ATAB could improve gut barrier functionality; this may contribute to the mechanisms underlying the therapeutic effects of ATAB on ALI.

To the best of our knowledge, this is the first study to explain the role of the gut microbiota during ATAB therapy for APAP-induced ALI. However, this study has certain limitations. First, ATAB is a monoclonal anti-TLR4 antibody, and its effects were most likely mediated through the antagonism of TLR4. Our study lacked treatment of APAP-treated mice with an isotype-matched control antibody, so we cannot rule out off-target effects in this study. Second, the causal relationship between the gut microbiota and the therapeutic effect of ATAB was analyzed insufficiently; for example, we did not perform research into specific bacterial strains. Different bacterial strains between the two groups should be screened and their functions should be verified in APAP-induced ALI. For instance, previous research has shown that Lactobacillus acidophilus LA14 could alleviate liver injury (42). Regarding the metabolites, we verified the function of SCFAs in ALI; however, we observed only that they can alleviate ALI, and the exact mechanism is still unclear and requires further research. Third, ATAB can improve gut barrier function; however, the molecular mechanism is unknown. Gut barrier function requires further analysis because of its importance in liver disease.

In conclusion, we reported a new therapeutic method for APAP-induced ALI in a mouse model and analyzed the role of the gut microbiota in this therapy. The fundamental data provided in this study may provide the foundation for further studies in this research area, leading to the development of new therapeutic strategies to treat APAP-induced ALI.

## MATERIALS AND METHODS

### Mouse model and treatment.

Male C57BL/6 mice (aged 4 to 6 weeks) were obtained from Sibeifu Biotechnology Company Limited (Beijing, China). The mice were housed in a specific-pathogen-free animal experiment facility with a temperature of 23°C ± 1°C, 53% ± 2% humidity, and a 12-h light/dark cycle. The mice were fed a standard laboratory diet (*ad libitum*) in individual standard stainless steel cages. To eliminate sex as an influencing factor in our experiments, we used only male C57BL/6 mice in this study. Therefore, it is important to bear in mind that the results for female mice may be different. All animals were fed adaptively for 1 week before the experiment. The mice were randomly assigned to three groups (APAP group, ATAB group, and control group [*n* = 8 for each group]). The animals in the APAP group were intraperitoneally injected with 600 mg/kg acetaminophen dissolved in PBS, the animals in the ATAB group were intraperitoneally injected with 5 mg/kg ATAB 2 h after acetaminophen injection, and the animals in the control group were intraperitoneally injected with the same volume of PBS. For antibiotic treatment, mice received vancomycin (100 mg/kg), neomycin sulfate (200 mg/kg), metronidazole (200 mg/kg), and ampicillin (200 mg/kg) intragastrically once daily for 5 days. Mice were not fasted before APAP treatment and were sacrificed 24 h after APAP treatment (56, 57).

The mouse experiments were carried out in accordance with the recommendations of the ethics provision for experiments on mice of the ethics committee of the Centre for Diseases Prevention and Control of Eastern Theater. The protocol was approved by the ethics committee of the Centre for Diseases Prevention and Control of Eastern Theater.

### Determination of biochemical parameters of serum and tissues.

The serum ALT and AST levels were determined using a detection kit (Jiancheng Bioengineering, Nanjing, China). Total SOD (T-SOD), MDA, GSH, and CAT levels in liver tissues and zonulin levels in colon tissues were determined using a detection kit (Jiancheng Bioengineering, Nanjing, China). The serum LPS level was measured with a detection kit (Jiancheng Bioengineering, Nanjing, China) according to the manufacturer’s instructions.

### Real-time fluorescence quantitative PCR experiments.

Total RNA was extracted from tissues using an RNA extraction kit. (Fastagen, Shanghai, China) according to the manufacturer’s instructions. Next, the total RNA concentration was determined by using a visible spectrophotometer, and reverse transcription was then used to synthesize cDNA. Real-time PCR was carried out on an ABI 7500 real-time PCR system. The real-time quantitative PCR (RT-qPCR) primers are shown in Table 1.

**TABLE 1:** RT-qPCR primer pairs

Target	Primer direction, sequence (5′–3′)
IL-6	Forward, TGATGCACTTGCAGAAAACA
	Reverse, ACCAGAGGAAATTTTCAATAGGC
CCL2	Forward, CCTGCTGTTCACAGTTGCC
	Reverse, ATTGGGATCATCTTGCTGGT
TNF-α	Forward, ACGTCGTAGCAAACCACCAA
	Reverse, TAGCAAATCGGCTGACGGTG

### Morphological analysis.

Tissue was collected and fixed in 4% paraformaldehyde. The sample was then dehydration and embedded (in paraffin), sliced, and stained with hematoxylin and eosin (HE).

### Fecal microbiota transplantation.

The mice were randomly assigned to four groups (ATAB group, APAP group, ATAB.R group, and APAP.R group [*n* = 6 for each group]). The ATAB.R and APAP.R groups received antibiotics intragastrically once daily for 5 days to deplete the gut microbiota. The feces of the donor mice (APAP group and ATAB group) were collected and resuspended in PBS at 0.125 g/mL. A total of 0.15 mL was administered to the ATAB.R and APAP.R groups. After 3 days, mice were intraperitoneally injected with 600 mg/kg APAP and sacrificed 24 h after APAP treatment.

### Microbial analysis.

Fresh fecal samples were collected using a metabolic cage and stored at −80°C. The fecal contents were resuspended in PBS (pH 7.4) containing 0.5% Tween 20, and the fecal suspension was stirred with a stirrer to destroy the bacterial membrane. The samples were sequenced on the Illumina Novaseq platform according to the manufacturer’s recommendations. It mainly includes DNA extracted from fecal contents for the amplification of variable region 4 (V4) of the bacterial 16S rRNA gene by PCR. Next, the product was purified, and the library was prepared and sequenced. A *t* test, a Wilcox rank sum test, and a Tukey test were used for data processing to analyze the differences between alpha diversity indices and beta diversity indices of the groups. Other charts were implemented by using the R package.

### Metabolomics analysis.

The metabolites were extracted from feces, the supernatant was collected, and the sample was injected for LC-MS analysis. The chromatographic column used was a Hypersil gold column (100 by 2.1 mm, 1.9 μm). The column temperature was 40°C, the flow rate was 0.2 mL/min, and the injection volume was 2 μL. Mobile phase A was 0.1% formic acid, and mobile phase B was methanol. Gradient elution was performed as follows: 0 to 1.5 min, 98% mobile phase A and 2% B; 1.5 to 3 min, 98% A and 2% B; 3 to 10 min, 0% A and 100% B; 10 to 10.1 min, 98% A and 2% B; and 10.1 to 13 min, 98% A and 2% B. The specific conditions for mass spectrometry were as follows: a spray voltage of 3.5 kV, a sheath gas flow rate of 35 arb (arb is a unit of measure of the gas flow rate), an auxiliary gas flow rate of 10 arb, a capillary temperature of 320°C, an S-lens radio frequency (RF) level of 60, and an auxiliary gas heater temperature of 350°C.

### Intestinal permeability.

To determine intestinal mucosal barrier permeability, 20 h after APAP injection, mice were given 4-kDa FITC-dextran (FD4) (500 mg/kg of body weight) (Sigma) orally. After 4 h, blood samples were collected from the orbit, and sera were separated. The fluorescence intensity of glucose molecular protein in peripheral blood was measured using a full-wavelength automatic enzyme labeling instrument (Tecan Spark) (excitation wavelength, 485 nm; emission wavelength, 525 nm). Paraffin sections of mouse colon specimens were dewaxed and dehydrated. The fluorescence intensity of colon tissue was observed by using a fluorescence microscope (Olympus, Japan).

### Application of SCFAs.

The mice were randomly assigned to three groups (APAP group, SCFA group, and control group [*n* = 8 for each group]). For the SCFA group, a short-chain fatty acid mixture (67.5 mM sodium acetate, 40 mM sodium propionate, 25.9 mM sodium butyrate) was dissolved in water and given to mice for free drinking for 7 days; next, APAP (600 mg/kg) was injected. For the APAP group and the control group, the experimental methods were the same as the ones described above. The blood and liver tissues of mice were collected after 24 h.

### Statistical analysis.

Data are expressed as means ± standard errors of the means (SEM). Unpaired two-tailed Student’s *t* test was used to assess differences between two groups. Data sets involving more than two sets were evaluated using a Newman-Keuls test. MetaX software was used to process the metabolomics data and for multivariate statistical analysis. Correlation analysis for different metabolites (Pearson correlation coefficient) was carried out in R (GraphPad Prism version 5.0; GraphPad Software). A *P* value of <0.05 was considered statistically significant.

### Data availability.

The 16S rRNA gene sequencing data are available at the NCBI Sequence Read Archive (SRA) database under BioProject accession number PRJNA826304.
