# Supplementary material for: Retracted and Republished from: “Gut Microbiota Mediates the Therapeutic Effect of Monoclonal Anti-TLR4 Antibody on Acetaminophen-Induced Acute Liver Injury in Mice”
Source: Microbiol Spectr. 2023 Mar 21;11(2):e04715-22. doi: 10.1128/spectrum.04715-22 (PMC10186863; doi:10.1128/spectrum.04715-22)
Supplement: Supplemental file 1 — Supplemental materials and methods and Fig. S1 and S2. Download spectrum.04715-22-s0001.pdf, PDF file, 0.3 MB [file spectrum.04715-22-s0001.pdf]

Supplement materials and methods

Male C57BL/6 mice (age, 4-6-weeks) were obtained from biotechnology limited company of SIBEIFU (Beijing, China). The mice were housed in a specific pathogen-free animal experiment facility at 23 ± 1 °C and 53 ± 2% humidity with a 12-h light/dark cycle. The mice were fed a standard laboratory diet (ad libitum) in individual standard stainless steel cages. All animals were fed adaptively for one week before the experiment. The mice were randomly assigned into three groups (APAP 6h group, APAP 24h group, and control group, n=4 each group). The APAP 6h group and APAP 24h group were intraperitoneally injected with 600 mg/kg acetaminophen dissolved in PBS, the control group were intraperitoneally injected with the same volume of PBS. The feces were collected at the beginning of the experiment (control group), at 6 hours (APAP 6h group) or 24 hours (APAP 24h group) after APAP treatment.

Figures:

Figure S1:

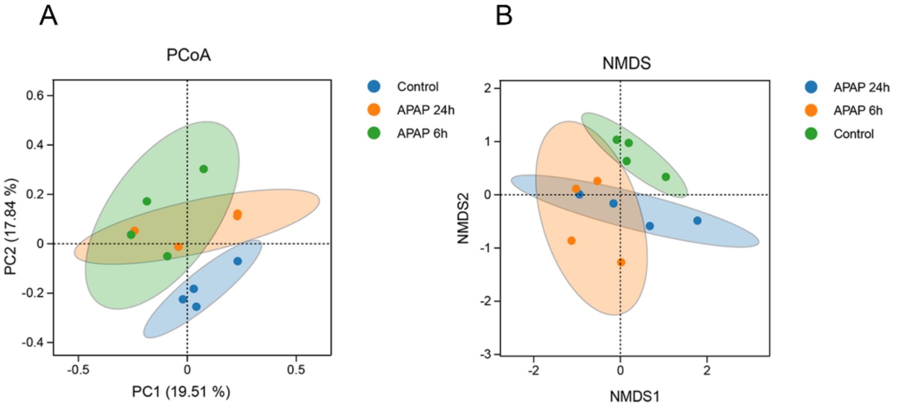

Figure S2:

A

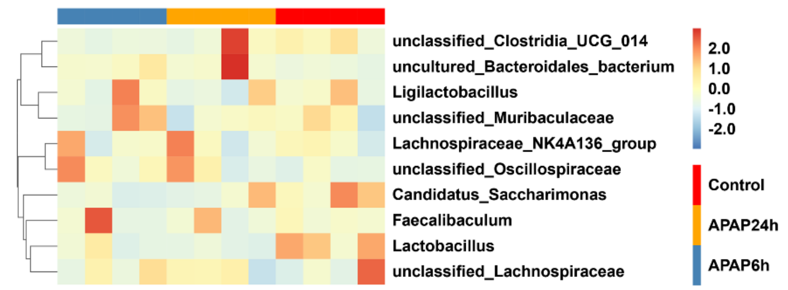
unclassified_Clostridia_UCG_014
uncultured_Bacteroidales_bacterium
Ligilactobacillus
unclassified_Muribaculaceae
Lachnospiraceae_NK4A136_group
unclassified_Oscillospiraceae
Candidatus_Saccharimonas
Faecalibaculum
Lactobacillus
unclassified_Lachnospiraceae
2.0
1.0
0.0
-1.0
-2.0
Control
APAP24h
APAP6h

B

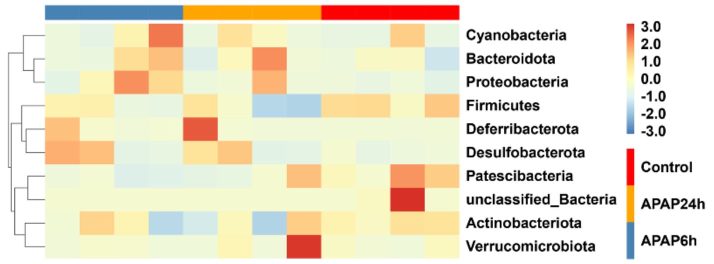
Cyanobacteria
Bacteroidota
Proteobacteria
Firmicutes
Deferribacterota
Desulfobacterota
Patescibacteria
unclassified_Bacteria
Actinobacteriota
Verrucomicrobiota
3.0
2.0
1.0
0.0
-1.0
-2.0
-3.0
Control
APAP24h
APAP6h

Figure Legends:

FIG S1: The gut microbiota was continually changing after APAP overused. (A-B) The PCoA and NMDS analysis of the gut microbiota. These results show a significant difference between APAP 6h group, APAP 24h group, and the control group. n=4 for each group.

FIG S2: The heatmap of the gut microbiota after APAP overused. (A-B) The heatmap on genus and phylum level of APAP 6h group, APAP 24h group, and control group. n=4 for each group.
